# Loneliness is associated with depression, anxiety, pain, and suffering in fibromyalgia: results from a structural equation modeling analysis

**DOI:** 10.1007/s00296-025-06036-6

**Published:** 2026-01-27

**Authors:** Juan Pablo Román-Calderón, Camila Andrea Sánchez Salazar, José Hugo Arias Botero, Alicia Krikorian

**Affiliations:** 1 EAFIT University, Medellín, Colombia; 2 Faculty of Medicine, CES University, Medellín, Colombia; 3 Pain and Palliative Care Group, School of Health Sciences, Universidad Pontificia Bolivariana, Cq.1 No. 70-01, Medellín, Colombia

**Keywords:** Fibromyalgia, Pain, Chronic pain, Psychological distress, Anxiety, Depression, Loneliness

## Abstract

Fibromyalgia is a widespread chronic pain condition affecting quality of life. Emotional distress and loneliness are factors commonly related to pain and suffering. However, no study has analyzed how all these variables interplay in individuals living with fibromyalgia. The study objective was to empirically test the relationships among loneliness, anxiety, depression, pain, and suffering in this population. Descriptive, observational, cross-sectional study. Structural equation modeling (SEM) was used to assess the relationships between loneliness, anxiety, and depression to the experience of pain and suffering in 317 fibromyalgia patients who attended a specialized pain management facility. Loneliness was positively associated with anxiety, pain, depression, and suffering. Moreover, in our sample, anxiety, depression, suffering, and pain demonstrated significant covariation. We also found that depression is positively related to pain and suffering. Our study demonstrates that loneliness in fibromyalgia is closely linked to anxiety, depression, pain, and suffering. Regular, comprehensive interventions that incorporate social support strategies may help reduce this burden.

**Supplementary Information:**

The online version contains supplementary material available at 10.1007/s00296-025-06036-6.

## Introduction

Fibromyalgia is characterized by widespread pain, fatigue, sleep disturbances, and functional symptoms, often overlapping with multiple comorbid symptoms [1]. Central sensitization, as well as inflammatory, immune, endocrine, genetic, and psychosocial factors, are involved in its pathogenesis. It is currently considered a multifactorial disease with physiological and somatic manifestations and significantly contributes to patient distress. Its reported prevalence in the general population ranges from 0.2 to 8% [2]. It is the most prevalent chronic widespread pain disease in clinical medicine and, as such, is considered by the World Health Organization to be a public health concern [1].

Emotional distress and psychiatric conditions are frequent in Fibromyalgia patients. The lifetime prevalence of depression and/or major depressive disorder ranges from 52 to 65%. It is nearly 33% for other psychiatric conditions, such as bipolar disorder, panic disorder, anxiety disorder (not otherwise specified), and posttraumatic stress disorder (PTSD) [3, 4]. Moreover, suicidal ideation is frequent and commonly related to emotional distress [5].

Additionally, loneliness, the subjective experience of social isolation and disconnection from the interpersonal intimacy and relationships one desires, and social isolation, including objective markers such as diminished social interactions and lack of social networks, are common in Fibromyalgia [6]. Both put individuals at risk for poorer health [7, 8]. Loneliness is currently considered an important risk factor for health problems [7]. It has been found to contribute to a cluster of pain, fatigue, and depression in adults in the general population [9]. Chronic loneliness has been linked to negative consequences for psychological and physical health, including increased risk for depression, anxiety, stress, stress-related inflammation, sleep disturbance, poorer immune functioning, and elevated cardiovascular risk [7, 10–12].

Furthermore, chronic loneliness significantly predicts subsequent physical and emotional health, even when accounting for depression and social support [10, 13]. Both chronic loneliness and daily fluctuations in loneliness predict emotional and physical health [14]. However, loneliness in fibromyalgia has received limited attention in research.

Specific links between loneliness and pain and between loneliness and other variables have also been reported [9, 14–17]. In a large cohort of older adults, Smith et al. reported that patients with musculoskeletal pain had a greater chance of experiencing loneliness [16], which was also associated with age, occupation, physical activity, and depression. Another population-based study in Japan revealed that loneliness was positively related to the occurrence of pain, its intensity, and the prevalence of past and present chronic pain [17]. According to the results of a study conducted by Wilson et al. in individuals with chronic pain, greater feelings of loneliness during the COVID-19 pandemic were associated with greater levels of pain catastrophizing, a relationship mediated by depression [14].

As mentioned, Fibromyalgia not only interferes with quality of life and daily functioning but is also associated with stress, psychological trauma, emotional distress [18], and loneliness [6], causing great suffering and threatening the person´s self and identity. Cassel defines suffering as “a specific state of severe distress associated with events that threaten the intactness of the person” [19]. Accordingly, Paschali et al. [20] found significant levels of threat to the self in Fibromyalgia patients using the Pictorial Representation of Illness and Self-image (PRISM), a common instrument used to assess suffering due to illness. Additionally, interest in recognizing, assessing, and approaching patient suffering and its related factors is rising [21, 22]. Thus, understanding the relationships among these variables is relevant to improving treatment options for Fibromyalgia.

In the context of a larger study examining the impact of Fibromyalgia on psychosocial problems, we decided to empirically test the relationships among loneliness, anxiety, depression, pain, and suffering. These relationships have not been studied altogether in Fibromyalgia, and, when little knowledge is available about potential causal links, it is recommended to establish if the variables of interest covary as a first step [23]. Testing these relationships altogether can help empirically map the nomological network of loneliness in Fibromyalgia. We decided to explore in a single statistical model the relationships of loneliness with the aforementioned covariates, and therefore we hypothesize that in Fibromyalgia: h1: Loneliness and anxiety are positively correlated; h2: Loneliness is positively correlated with depression; h3: Loneliness and pain are positively correlated; and h4: Loneliness is positively correlated with suffering.

Although the focus of our study was the covariance of loneliness with anxiety, depression, suffering, and pain, we also included the relationship between anxiety, depression, suffering, and pain, since it has been reported in previous research. For instance, prior literature suggests that depression and anxiety [20, 24] and suffering and pain [20] are associated.

## Materials and methods

We conducted a descriptive, observational, cross-sectional study. Cross-sectional designs are recommended when research is in exploratory stages and scarce empirical evidence about time frames in causal relationships is available [23].

### Participants and procedures

Patients who were diagnosed with Fibromyalgia using current diagnostic criteria by attending specialists in rheumatology or pain and palliative care, whose main complaint was this condition, and who were treated at a specialized pain management center in Medellín, Colombia, were invited to participate. The inclusion criteria were being an adult older than 18 years and having ongoing treatment for Fibromyalgia. Patients were excluded if they had cognitive or language limitations in comprehending and responding to questionnaires; a pain crisis; or comorbidities such as cancer, multiple sclerosis or other demyelinating diseases; dementia; or other neurodegenerative, infectious, traumatic, or surgical pathologies.

We calculated a sample size of 292 patients based on a prevalence of suffering of 75% based on a previous study [20], a confidence of 95%, and a precision of 5%. We used a convenience sample technique, and all patients who attended consultations between July 2020 and August 2021 and who were eligible were invited to participate. The study was performed in line with the principles of the Declaration of Helsinki and was approved by the ethics committee of the Universidad Pontificia Bolivariana (record 10-2021, dated May 24, 2021) and the ethics committee of Incodol (IN-CE1-02/2020, dated September 18, 2020).

### Instruments

After patients signed the participation consent, sociodemographic and clinical data were collected from clinical charts. Trained healthcare staff conducted structured in-person interviews during follow-up consultations that included the administration of a set of assessment tools:

We assessed suffering using the PRISM task [25]. We selected the electronic version of the PRISM, which has been previously used in studies to evaluate illness-related suffering [26]. PRISM is a valid and reliable graphic strategy for assessing and quantifying qualitative issues and has been widely utilized to assess suffering in our context [27–29]. Patients were shown a white panel (210 × 297 mm) corresponding to the display of the tablet, with a yellow circle (diameter of 70 mm) at the bottom right-hand corner. Patients were asked to imagine that the panel represented their life at this moment in time, with the yellow circle representing “themselves”. Next, they were asked to imagine that another smaller red circle (diameter of 50 mm) stood for their fibromyalgia and were asked the following question: “Where would you place the fibromyalgia-circle in your life at the moment?” The self-illness separation (SIS) score, defined as the distance between the centers of the two circles (ranging from 0 to 25.6 cm), is used to quantify suffering. A greater distance indicates less suffering due to illness. To ease the interpretation of our results, we reversed the raw scores, with higher scores indicating higher suffering due to illness.

Pain was assessed using the visual analog scale (a 0 to 10 scale) of the Fibromyalgia Impact Questionnaire [30] in its Spanish validation [31].

We evaluated loneliness using the University of California Loneliness Scale (UCLA), a widely used scale for loneliness assessment [32, 33]. We used the 10-item version validated in Spanish [34]. Participants tap their responses using a 4-point Likert scale, with scores ranging from 10 to 40, where lower scores indicate more loneliness, and scores below 31 indicate clinical states of loneliness. The scale has shown very good internal consistency (α = 0.95). For our study, we used raw scores.

We measured anxiety and depression using the Hospital Anxiety and Depression Scale (HADS), a widely used measure to assess illness-related anxiety and depression symptoms [35]. HADS comprises 14 items, half of which measure anxiety (HADS-A) and the other half of which measure depression (HADS-D), with a cutoff point of 8 for the anxiety subscale and 9 for the depression subscale. Higher scores indicate greater severity. HADS has also been used to assess emotional distress in Fibromyalgia [36]. For the current study, we used a validated version for the Colombian population [37]. It is important to note that researchers deleted items 8 and 9 to maintain the two-factor structure, resulting in a final HADS version of 12 items.

Since we only administered self-report measures, we applied other procedural remedies to address common method bias. Following Podsakoff et al. [38], we introduced methodological separation in the measurement of the predictor and one of the criterion variables (i.e., suffering). We used verbal paper questionnaires and a visual computer-assisted scale.

### Data analysis

Unlike traditional correlation analyses, which test associations between composite scores, structural equation modeling (SEM) enables us to examine the relationships between latent constructs. Furthermore, the covariance-based (SEM) technique SEM helped us to test the hypothesized relationships while accounting for measurement error. SEM allowed us to include PRISM and Pain, single-item measures, as observed variables in our hypothesized model. Otherwise, modelling PRISM and Pain as indicators of two additional latent variables would have implied corrections of residual variances. We analyzed our data using the Mplus statistical package [39]. In line with the hypotheses of our study, we tested a set of Confirmatory Factor Analysis (CFA) models with covariates. The maximum likelihood with robust standard error (MLR) estimator allowed us to continue our analyses despite some violations of the normality assumption.

We used the chi-square (χ^2^), the comparative fit index (CFI), and the root mean square error of approximation (RMSEA) to assess the goodness of fit of our models, and the Satorra-Bentler chi-square difference test (χ^2diff)^ to compare the CFA models [40]. A χ^2^ with a p value>0.05 suggested a good model fit. We adopted the cutoff values suggested by Hu and Bentler [41] for the CFI and RMSEA. CFI values close to .95 and RMSEA values close to 0.06 are indicative of a good model fit. Concerning the factor loadings, since we had a sample with more than 250 observations, we used < 0.35 as the critical value [34].

Although these coefficients are largely used, the validity of traditional rules of thumb has been questioned. As a remedy for the inaccuracy of traditional cut of values, in addition to observing the goodness of fit of SEM models, researchers should test competing models and evaluate model local fit [42]. Although there is an important consensus of a two-factor HADS structure (i.e., anxiety and depression), some studies suggest that the items may measure a single distress factor [43]. Therefore, as a competitive model, we tested a CFA where HADS items loaded on one factor. To test local fit, we examined the residual correlation matrix, looking for values .20 above the residual correlation mean (Q3max). Such values are indicative of local dependency [44]. Finally, with the retained items, we calculated McDonald’s Omega to account for the reliability of the measures of latent variables.

## Results

### Descriptive statistics

A total of 329 patients were invited to participate in our study, and 317 of them completed the instruments. A total of 96.8% of the participants were women, with an average age of 53 years; 53% of them were married or living with a partner, and a median of 3 people lived in the household. They had a median of five years since diagnosis (IQR3-10) and a median of two comorbidities (IQR 1-4). Most of them received analgesics, and other common treatments included anticonvulsants, antidepressants, psychiatric treatment, and physiotherapy. All of them received standard treatment that included a multimodal approach. More than half did not perform physical activity or did so occasionally (less than three days a week) (Table 1).

**Table 1:** Sociodemographic and clinical characteristics (n = 317)

Variable	Median	Interquartile range	Min–max
Number of people living in the household	3	(2–4)	0–9
Years since diagnosis	5	(3–10)	0–30
Number of consultations in the last month	0	0	0–10
Number of comorbidities	2	(1–4)	0–10

Variable	Category	n (%)
Sex	Women	307 (96.8)
Civil status	Single	70 (22.1)
	Married/free union	169 (53.3)
	Divorced	49 (15.5)
	Widowed	29 (9.2)
Residence	Urban	276 (87.1)
Education level	No studies	6 (1.9)
	Primary school	68 (21.5)
	High school	125 (39.4)
	College/university	114 (36.0)
	Graduate studies	4 (1.3)
Occupation	Employee	91 (28.7)
	Independent	36 (11.4)
	Housewife	118 (37.2)
	Unemployed	18 (5.7)
	Student	2(0.6)
	Retired	52 (16.4)
Needs caregiving	No required	286 (90.2)
	Occasionally	10 (3.2)
	Frequently	11 (3.5)
	Permanently	3 (1.0)
	Required, but not available	7 (2.2)
Physical activity	Never	72 (22.7)
	Very occasional (1–4 times a month)	76 (24)
	Occasional (5–8 times a month)	39 (12.3)
	Frequent (3–5 times a week)	106 (33.4)
	Always (6–7 days a week)	24 (7.6)
Treatments received	Analgesics	306 (96,5)
	Anticonvulsants	149 (47)
	Antidepressants	144 (45,4)
	Psychiatric treatment	145 (45,7)
	Psychotherapy	100 (31,6)
	Family therapy	8 (2,5)
	Physiotherapy	123 (38,8)
	Complementary treatment	77 (24,3)

### Structural equation model

As shown in Table 2, the initial CFA model we specified (CFASPEC), considering the complete scales and hypothesized covariates, obtained χ^2^ and CFI marginal scores. Since the validity of the traditional rules of thumb has been questioned, we proceeded to check the residual correlations matrix to identify sources of local misfit affecting the CFASPEC general results. We found that the residual correlation between items UCLA 9 and UCLA 10 was 0.207 and higher than the Q3mx value (0.198). Item UCLA 9 showed a high residual correlation with item UCLA 7 (0.194). The Mplus output of CFASPEC, including the residuals correlation matrix, is included in Online Appendix A. Therefore, we dropped item UCLA 9 and, after the examination of local fit, we fitted a new CFA (CFALOCALFIT). The CFALOCALFIT showed a better general fit (i.e., CFI and RMSEA). The Mplus syntax and output of the CFALOCALFIT model are included in Online Appendix B. We proceeded to compare this model with a CFA with a single distress factor, HADS (CFADISTRESS), instead of a two-factor (anxiety and depression) HADS. We report the Mplus syntax and output of the CFADISTRESS model in Online Appendix C. The general fit worsened, and this was confirmed by the χ^2diff^ test (Trd = 107.25). The latter suggested that the model with more free parameters, CFALOCALFIT, should be retained.

**Table 2:** Goodness-of-fit and difference test

Competing models	χ2	df	p	cd	CFI	RMSEA	χ2 diff
CFASPEC	440.72	244	< 0.05	1.058	0.913	0.05	
CFALOCALFIT	370.76	222	< 0.05	1.056	0.931	0.046	
CFADISTRESS	478.01	226	< 0.05	1.056	0.883	0.059	107.25

*χ2* Chi-square, *df* Degrees of freedom, *cd* Scaling correction factor, *CFI* Comparative fit index, RMSEA Root mean square error of approximation, *χ2diff* Chi-square difference test

**Fig. 1:**
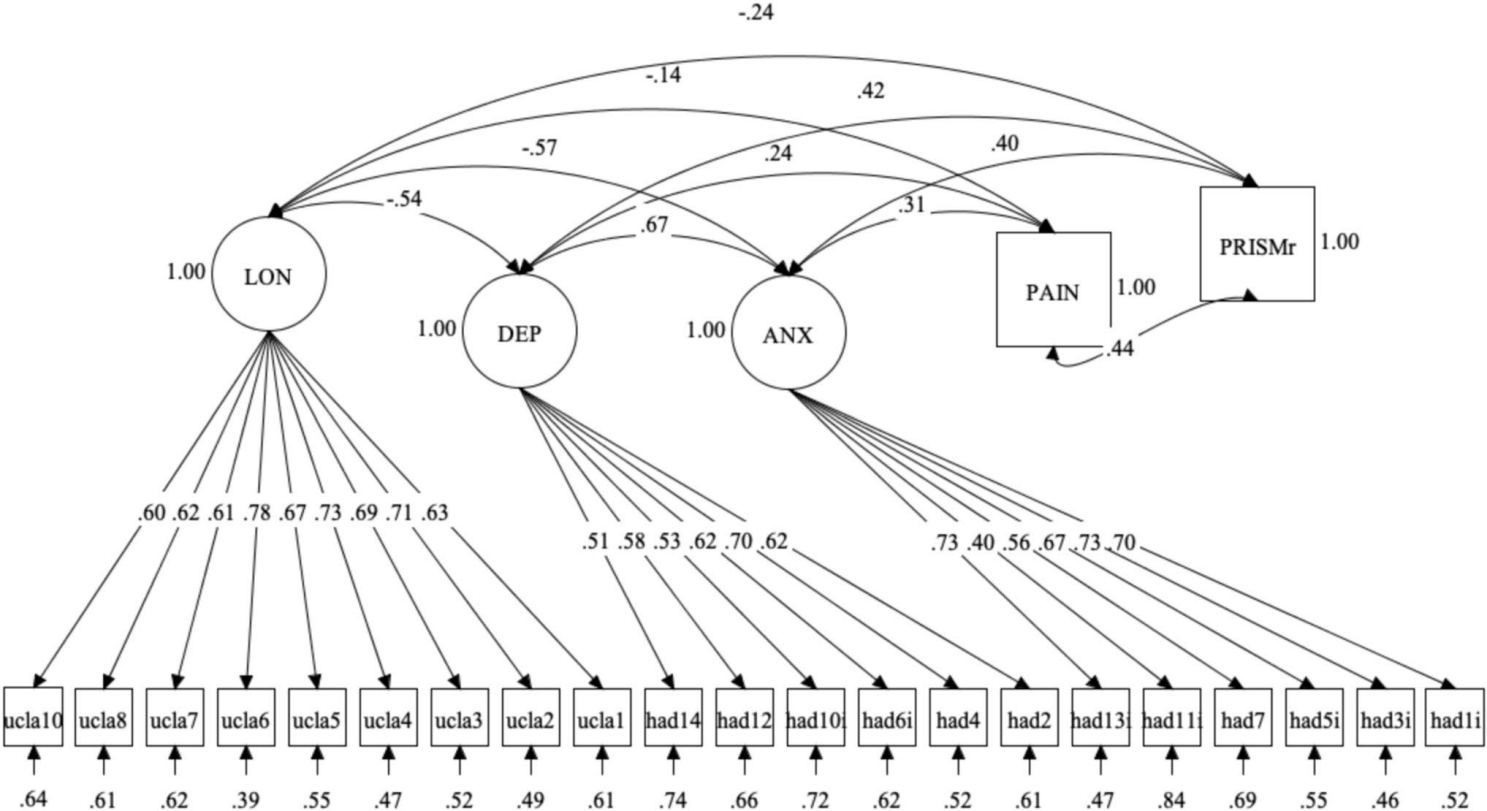
Retained CFA with covariates of loneliness. Mplus diagrammer output standardized results. *N* = 317; Estimator = MLR; Factor variances are fixed to 1, and covariances equal correlations between factors. All parameters are significant at the *p* < 0.01 level, except for the correlation between PRISM and LON, significant at the *p* < 0.05 level.

Figure 1 displays the specific results of the CFALOCALFIT with covariates. The associations between UCLA with pain, anxiety, and depression resulted negative (as loneliness increased, also pain, anxiety, and depression increased), and the relationship of UCLA with the reversed scores of PRISM (suffering) also resulted negative (indicating that, as loneliness increased, also did suffering due to illness). Considering that the UCLA scale is reversed, our results support our hypotheses according to which a higher loneliness in Fibromyalgia is associated with higher anxiety, depression, pain, and suffering. As expected, we also found that higher anxiety is associated with higher depression. Also, the associations of PRISM reversed scores with pain, anxiety, and depression resulted positive, indicating that higher suffering is associated with higher pain, anxiety, and depression. To facilitate the interpretation of these results, we report the estimated correlation matrix for latent variables in Table 3.

**Table 3:** Estimated means and correlation matrix, and composite reliability

Latent variable	Mean	1	2	3	4
1. ANX		**0.80**			
2. DEP		0.67**	**0.76**		
		[0.57 0.76]			
3. LON		− 0.57**	− 0.54**	**0.88**	
		[− 0.66 − 0.49]	[− 0.63 − 0.45]		
4. PRISM	6.51	0.40**	0.42**	− 0.24**	
		[0.31 0.49]	[0.33 0.51]	[− 0.34 − 0.15]	
5. PAIN	7.71	0.31**	0.24**	− 0.14*	0.44**
		[0.22 0.41]	[0.14 0.34]	[− 0.25 − 0.04]	[0.34 0.53]

Reversed scores for PRISM we used. PRISM and PAIN are single-item measures. **Significant at the *p* < 0.01 level; *Significant at the *p* < 0.05 level. Below correlations [LL UL] lower and upper limit 95% confidence intervals. Mc Donald’s omega in diagonal

In addition to the tests of our hypotheses, we conducted several post hoc analyses to test the robustness of our findings. Since we used a reduced version of the HADS scale validated in Colombia in cancer patients, we tested a CFA including all the HADS items and evaluated if there were changes in our findings. We did not find any changes in the direction or significance of the relationships between loneliness, depression, anxiety, pain, and suffering. We present the syntax and complete results of the model with all HADS items in Online Appendix D.

Finally, as a sensitivity analysis, we included possible confounders in an additional CFA. We added age, education, and physical activity as covariates of loneliness, depression, anxiety, pain, and suffering. The complete results of the CFA with these covariates are presented in Online Appendix E. We did not find any difference in the direction or significance in the relationships between loneliness, depression, anxiety, pain, and suffering. Loneliness was not significantly related to age (*r* = 0.11, *p* >0.05; 95% CI [0.01, 0.21]), education (*r* = 0.04, *p* >0.05; 95% CI [− 0.06, 0.15]), or physical activity (*r* = 0.12, *p* >0.05; 95% CI [0.02, 0.22]). Conversely, we found that physical activity was negatively associated with depression (*r* = − 0.25, *p* < 0.01; 95% CI [− 0.30, − 0.11]), anxiety (*r* = − 0.14, *p* < 0.05; 95% CI [− 0.30, − 0.11]) and suffering (*r* = − 0.13, *p* < 0.05; 95% CI [− 0.30, − 0.11]). We also found that age resulted negatively correlated with anxiety (*r* = − 0.21, *p* < 0.01; 95% CI [− 0.30, − 0.11]).

## Discussion

Our article sheds light on the role of loneliness, depression, anxiety, and suffering in individuals living with Fibromyalgia. These variables and their relationships are crucial to address in clinical practice because they significantly affect the overall well-being and quality of life of those living with Fibromyalgia. The primary objective of the study was to test a conceptual model that specifies the interrelationships among these variables, aiming to enhance our understanding of their complex dynamics. Our results show that loneliness is positively associated with anxiety, pain, depression, and suffering. Thus, they contribute to the theory by mapping the nomological network of loneliness in Fibromyalgia.

Although the interest in understanding the relationship between loneliness and emotional distress in Fibromyalgia is increasing, there is still a lack of comprehensive understanding of these matters. Suffering extends beyond the physical realm and encompasses emotional distress associated with the limitations imposed by the condition, including difficulties in maintaining employment, engaging in hobbies, and maintaining social relationships [45]. Fibromyalgia is a chronic condition characterized by widespread pain and fatigue [1] often accompanied by social isolation due to limitations in physical activities and participation in social gatherings [3]. Also, individuals with fibromyalgia face a lack of comprehension from their support group and even invalidation and discounting (responses of disbelief, criticism, dismissal of inability to work, not acknowledging symptom fluctuations, and offering unusable advice) [46]. The resulting sense of loneliness can intensify the psychological burden faced by individuals with Fibromyalgia [6]. In line with these assertions, we found that loneliness is positively associated with depression, anxiety, pain, and suffering in individuals living with Fibromyalgia. We also found in our sample that depression is positively related to pain and suffering. These results underscore the importance of addressing loneliness in patient care, highlighting the need for healthcare professionals to provide targeted support and interventions.

One possible explanation for the association between loneliness, depression, and pain is the societal invalidation of pain or other symptoms [46], deficits in positive emotions, and poor-quality social connections [6, 47]. Previous studies have concluded that individuals with chronic pain who experience chronic loneliness are more likely to report higher levels of interpersonal stress and experience increased daily pain due to the influence of depression [6].

Current evidence also suggests that, among healthy individuals, experiencing loneliness is associated with lower levels of perceived control and self-efficacy [48], as well as higher levels of rumination regarding a lack of control in stressful situations [49]. In the case of Fibromyalgia patients, loneliness is associated with more intense maladaptive pain cognitions, such as catastrophizing about pain, which in turn predicts higher levels of pain experienced in the evening [50]. Our findings show that there is an interplay between loneliness, anxiety, and pain. The literature suggests that loneliness may exacerbate anxiety, particularly when individuals feel a sense of lack of control in stressful situations, such as living with Fibromyalgia, and through engaging in catastrophizing thoughts about these symptoms. These results align with studies conducted on healthy individuals, which have linked loneliness to negative cognitions related to stress management [11].

It is important to note that this study was conducted during COVID-19, where social contact restrictions due to the pandemic may have exacerbated loneliness and social isolation, among other health variables. However, the evidence shows otherwise. A study conducted in Italy that examined changes in patient-reported outcomes before and after the lockdown period in fibromyalgia patients found mixed results on their well-being: while for some it resulted in increased severity, for others it brought beneficial changes and improvements in well-being [51]. Moreover, another study conducted in the Netherlands using a repeated cross-sectional design found a slight improvement in the health status of fibromyalgia patients before and after the pandemic, suggesting that some circumstances regarding COVID-19 were somewhat favorable for women with fibromyalgia [52].

In recent decades, the relevance of suffering has been increasingly recognized; however, there remains a knowledge gap that requires further exploration in chronic pain, particularly in the case of Fibromyalgia. This medical condition is one of the most prevalent and costly globally [1] and is closely associated with suffering [20]. We found in our sample of individuals living with Fibromyalgia that pain and suffering are positively associated, therefore contributing to a limited body of literature that comprehensively examines this connection [53].

Addressing the challenges regarding loneliness, suffering, and emotional distress in Fibromyalgia patients requires a comprehensive multidisciplinary approach. This is relevant given that social variables (such as social support) remain uninvestigated and, thus, undertreated [54]. Healthcare providers should prioritize not only the management of physical symptoms but also the social and psychological well-being of individuals with Fibromyalgia, implementing regular assessment and treatment strategies to mitigate them, such as support groups, counseling, and fostering social connections among patients [55]. Furthermore, mental health support, such as therapy and counseling, should be integrated into the treatment plan for Fibromyalgia patients.

According to our analyses in terms of measurement, in addition to the items deleted in the Colombian version of the HADS, we had to drop an item from the UCLA scale. These results have important implications. Researchers should be careful when reporting the prevalence of latent variables in their samples after factor analyses, implying the deletion of items. Minimum, maximum, and aggregate values coming from sums of item scores are affected by the deletion of indicators.

Our study had some limitations to consider. Despite our efforts to use different types of measures to reduce common method bias, the data collected were based on self-reports. Future studies should include other assessment strategies that allow for greater objectivity of the collected information. For instance, researchers could also assess loneliness using information collected from raters such as family members or friends. Although such information may help to contrast patients’ self-reports of loneliness, we believe that information based on the patient's perception should not be underestimated, especially for painful pathologies where patient-reported outcomes have gained great importance.

Another limitation of our study lies in its cross-sectional design. Cross-sectional designs allow for observation of only the studied variables at a given moment and their relationship without establishing causality. Our results help map the nomological network of loneliness in individuals living with Fibromyalgia. This is a necessary step before claiming empirical evidence of the relationship between loneliness, depression, anxiety, suffering, and pain. Despite prior assertions about the nature of these relationships, no cross-sectional study could estimate these effects. Testing the effects of loneliness on the variables included in its nomological network, aspects such as the time frame necessary for one variable to affect the others should be established. Once such aspects are clarified, future longitudinal studies could test, for instance, models in samples of individuals living with Fibromyalgia where the effect of loneliness on suffering and pain is mediated by anxiety and depression. Finally, data were collected in a single institution, limiting its generalizability. We recommend that future studies analyze data collected from patients from different institutions. Since health care institutions may implement different strategies to manage social and psychological well-being, such data could also be useful to test their effectiveness in the treatment of psychosocial symptoms in patients with fibromyalgia.

Despite these limitations, we believe that our findings stress the need for healthcare providers, researchers, and society to acknowledge and address the complex relationships among loneliness, suffering, emotional distress, and pain. These findings can serve as a foundation for further exploration of these variables, aiming to develop new therapeutic approaches that go beyond pharmacological management and encompass a more comprehensive approach to patient care, including social variables. They also add to the current evidence, integrating loneliness into the understanding of fibromyalgia´s associated distress. In fact, newly developed instruments to assess the quality of life in fibromyalgia patients include social variables such as social relationships and isolation, but not specifically loneliness [56]. Furthermore, as Willemse et al. state, understanding links between fibromyalgia severity and psychosocial processes may provide clues to increase the quality of life of persons with fibromyalgia [46]. Thus, this research sheds light on the contributions of loneliness to the overall suffering of fibromyalgia patients.

## Supplementary Information

Below is the link to the electronic supplementary material.

Supplementary file1 (XLSX 76 kb)

Supplementary file2 (XLSX 73 kb)

Supplementary file3 (XLSX 71 kb)

Supplementary file4 (XLSX 83 kb)

Supplementary file5 (XLSX 90 kb)

## Data Availability

Data are available on request.
